# The Possibility of Interlocking Nail Fabrication from FFF 3D Printing PLA/PCL/HA Composites Coated by Local Silk Fibroin for Canine Bone Fracture Treatment

**DOI:** 10.3390/ma13071564

**Published:** 2020-03-28

**Authors:** Siwasit Pitjamit, Kittiya Thunsiri, Wasawat Nakkiew, Tunchanok Wongwichai, Peraphan Pothacharoen, Wassanai Wattanutchariya

**Affiliations:** 1 Department of Industrial Engineering, Faculty of Engineering, Chiang Mai University, Chiang Mai 50200, Thailand; siwasit_p@cmu.ac.th; 2 Advanced Manufacturing Technology Research Center (AMTech), Department of Industrial Engineering, Faculty of Engineering, Chiang Mai University, Chiang Mai 50200, Thailand; kittiya.thunsiri@hotmail.com (K.T.); wassanai@eng.cmu.ac.th (W.W.); 3 Biomedical Engineering Institute, Chiang Mai University, Chiang Mai 50200, Thailand; 4 Thailand Excellence Center for Tissue Engineering and Stem Cell, Department of Biochemistry, Faculty of Medicine, Chiang Mai University, Chiang Mai 50200, Thailand; tunchanok.wong@gmail.com (T.W.); peraphan.pothacharoen@gmail.com (P.P.)

**Keywords:** 3D printing, biocomposite materials, biomedical applications, mechanical properties, biodegradable, cytotoxicity

## Abstract

The biomaterials polylactic acid (PLA), polycaprolactone (PCL), and hydroxyapatite (HA) were selected to fabricate composite filaments for 3D printing fused filament fabrication (FFF), which was used to fabricate a composite biomaterial for an interlocking nail for canine diaphyseal fractures instead of metal bioinert materials. Bioactive materials were used to increase biological activities and provide a high possibility for bone regeneration to eliminate the limitations of interlocking nails. HA was added to PLA and PCL granules in three ratios according to the percentage of HA: 0%, 5%, and 15% (PLA/PCL, PLA/PCL/5HA, and PLA/PCL/15HA, respectively), before the filaments were extruded. The test specimens were 3D-printed from the extruded composite filaments using an FFF printer. Then, a group of test specimens was coated by silk fibroin (SF) using the lyophilization technique to increase their biological properties. Mechanical, biological, and chemical characterizations were performed to investigate the properties of the composite biomaterials. The glass transition and melting temperatures of the copolymer were not influenced by the presence of HA in the PLA/PCL filaments. Meanwhile, the presence of HA in the PLA/PCL/15HA group resulted in the highest compressive strength (82.72 ± 1.76 MPa) and the lowest tensile strength (52.05 ± 2.44 MPa). HA provided higher bone cell proliferation, and higher values were observed in the SF coating group. Therefore, FFF 3D-printed filaments using composite materials with bioactive materials have a high potential for use in fabricating an interlocking nail for canine diaphyseal fractures.

## 1. Introduction

Diaphyseal fractures in dogs and cats are common small animal orthopedic injuries that can be treated by using interlocking nails. Interlocking nails were developed to increase treatment abilities, especially in canine femur, tibia, and humerus fractures [1]. However, the removal of interlocking nails is still required due to complications in long-term implantation [2]. Titanium and stainless steel are the biomaterials commonly used in interlocking nails. According to the bioinert properties of these materials, biological support to the living cells is not activated, and bone regeneration does not occur in the interlocking nail cavity [3]. Therefore, the interlocking nail will leave a blank cavity inside the healed bone. Tissue engineering has become necessary for bone treatment to eliminate the limitations of the natural healing process using several materials and fabrication processes [4,5,6]. Fused filament fabrication (FFF) is a well-known 3D printing method and an additive manufacturing (AM) technology that is widely used in several fields [7]. It provides several benefits, such as product customization, cost-effectiveness, and the possibility to create complex structures, making it ideal for patient-specific devices in biomedical applications [8,9]. To implement FFF for implantable biomedical devices, meltable biomaterial filaments are required [10,11]. Generally, meltable polymers are key materials for FFF 3D printing. Thus, the load carrying and mechanical properties of the polymers interlocking nails should be focused on. Although polymers provide low mechanical properties, they are still able to carry the mechanical load on small animal bones, such as feline and canine femurs [12]. Thus, we aimed to use an FFF 3D-printed interlocking nail for the treatment of canine orthopedic injuries. However, each individual biomaterial has its own disadvantages for tissue regeneration. Thus, copolymers or blended composite materials are required to improve the properties of the fabricated materials [13]. Currently, biomaterial filaments, such as polylactic acid (PLA) and poly-caprolactone (PCL), are commercially available for FFF 3D printing. PLA is widely used in biomedical applications because the compound released during its degradation (lactic acid) is a non-cytotoxic compound found in the mammalian body [14,15]. However, PLA is brittle and rapidly degraded in body fluids [16]. Therefore, blending PLA with other biodegradable polymers was performed to decrease the substance’s limitations [17,18]. PCL is a biodegradable polymer blended with PLA to decrease the brittleness problem and prolong the degradation period [18]. Furthermore, PLA and PCL are combined into a copolymer that provides the required abilities for tissue engineering applications, such as being environmentally friendly, non-toxic, biocompatible, and biodegradable [13,19]. To increase osteoconductivity and induce bone growth, hydroxyapatite (HA), the main mineral component in mammalian bone, has been used in several studies [20,21,22,23]. Moreover, a biomaterial with good osteoconductivity and osteoinductivity is silk fibroin (SF), which is commonly used in biomedical applications [24,25,26]. Thus, PLA, PCL, HA, and SF were selected for use in this study. The composite biomaterial filaments were locally extruded from three combination ratios between PLA, PCL, and HA. The filaments were then FFF 3D-printed into four design specimens for the different tests. Finally, SF was coated on the printed specimens using the lyophilization technique for cytotoxicity and cell proliferation tests. The mechanical and biological properties of the specimens were used to investigate the possibility to fabricate an interlocking nail using FFF 3D-printed PLA/PCL/HA composite filaments and coatings using local silk fibroin for the canine bone fracture treatment.

## 2. Materials and Methods 

Commercial PLA and PCL granules were used for filament extrusion. PLA is mainly composed of a semicrystalline poly-lactide resin (Ingeo 3D850, Natureworks LLC, Blair, Nebraska, USA) [27]. The PCL (number-average molecular weight (Mw) = 60,000–80,000 Da) was provided by Daigang Biomaterials (Shandong, China). PCL is a semicrystalline biodegradable hydrophobic polyester that is widely used in 3D printing [28].

HA was synthesized from bovine bone. After cutting into small pieces and soaked in H_2_O_2_ for 2 days to remove the tissues, the small pieces of bones were boiled in water to eliminate organic substances. Then, the boiled bones were dried in a hot air oven at 120 °C for 7 h to reduce their moisture. The dried bones were calcinated to approximately 850 °C for 3 h and ground to less than 20 microns by a high ball-milling machine [5,29]. HA powder was added to a silane coupling solution at a ratio of 1:2 w/v. Finally, the slurry HA was dried at 100–120 °C [30].

Local Thai *Bombyx mori* (Luang Saraburi) silk cocoons were used as raw materials for SF extraction. Each cocoon was cut small and degummed in 100 °C Na_2_CO_3_ solution for 30 min and rinsed with warm deionized (DI) water. The degummed silk fibers were dried overnight in a 37 °C hot air oven and then dissolved at 70 °C for 6 h in a ternary solvent (CaC_l2_/CH_3_CH_2_OH/H_2_O; 1:2:8 in mole ratio) [31,32]. The silk-ternary solution was dialyzed using a cellulose membrane with 4 °C DI water for 3 days, and the DI water was changed every day. The dialyzed SF solution was filtered centrifuged and frozen at −80 °C. Finally, the frozen SF was lyophilized to obtain SF sponges [6].

**Figure 1:**
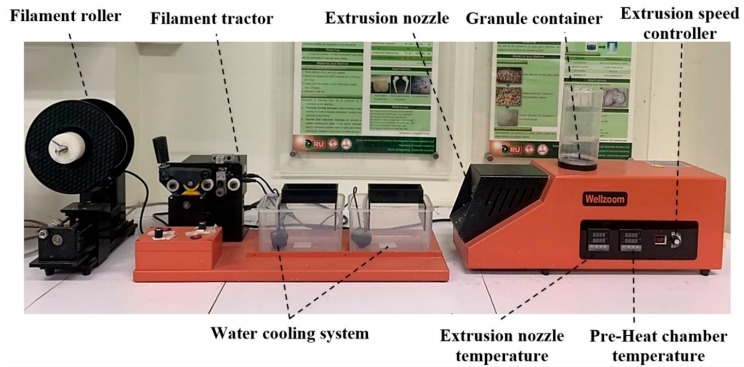
Wellzoom Desktop Extruder Line II.

The mixed materials were extruded to be a single filament using a desktop single-screw extruder (Wellzoom Desktop Extruder Line II, Shenzhen, China) with a filament cooling system, as shown in Figure 1. The mixing ratio between the PLA and PCL was fixed at 70:30 [33]. HA powder was added to the prepared PLA/PCL mixed granules at 0%, 5%, and 15% w/w. From our preliminary experiments, the maximum percentage of HA powder in the PLA/PCL mixed granules was limited by the ability of the extruder and the extruded filament conditions. Moreover, the maximum HA percentage from previous studies was presented at 15% [34,35,36]. The mixing ratios were labeled and are described in Table 1. The moisture in the PLA granules was reduced in a 60 °C oven for 4 h prior to mixing [37]. Then, PLA, PCL, and HA were homogenously mixed using a homemade ball milling machine (1440 rpm). The controlled extrusion parameters consisted of the extrusion rotational speed, the temperature of the pre-heated chamber, and the temperature of the extrusion nozzle, as presented in Table 2. The first run filament was tracked to the cooling system manually until the tip of the filaments reached the filament tractor. Then, the tractor speed was adjusted until the extruded filament diameter was 1.75 ± 0.05 mm and ran smoothly [38,39]. The filament was rolled into a filament spool using a filament roller, which adjusted the rolling speed based on the filament’s tractor speed.

The 200 mm long extruded filaments were clamped with tensile grips in a universal testing machine (UTM: Instron 5566, Ithaca, NY, USA). A 500 N loading cell was used with a displacement rate of 5 mm/min [40].

**Figure 2:**
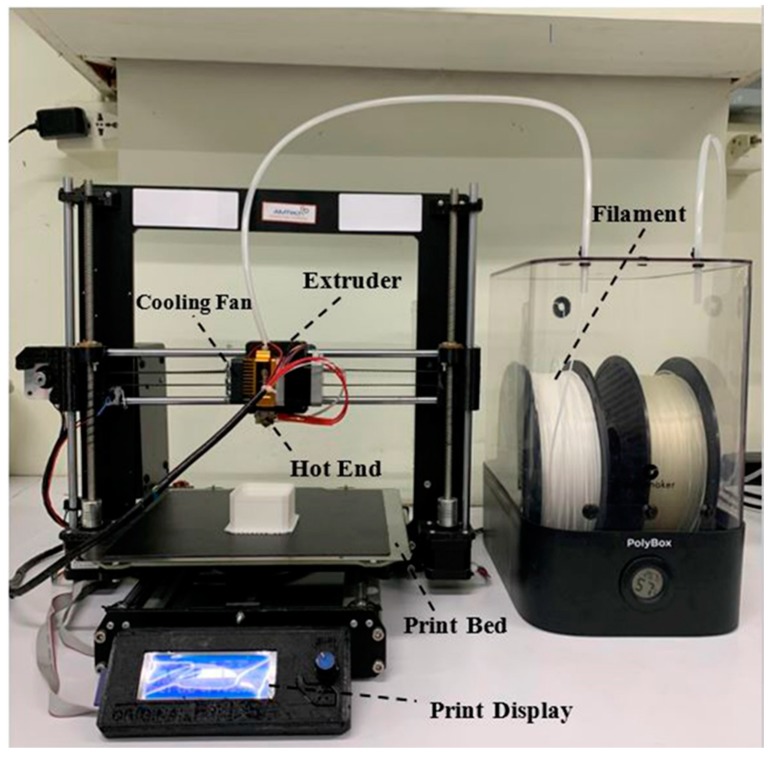
Homemade fused filament fabrication (FFF) 3D printer.

All specimens were printed using a homemade FFF 3D-printer in our laboratory, as shown in Figure 2. A 0.4 mm printing nozzle was used. The printing parameters were implemented via a computer program for 3D-printing. The melting temperature of the printing nozzle was 200 °C, the extrusion speed was 50 mm/s, and the printing layer height was 0.2 mm [41]. The printed specimens were designed based on mechanical standard testing and previous publications.

Cylindrical specimens 10 mm in diameter and 10 mm in thickness were printed for the compression test. Each specimen was placed in the compressing station of an Instron 5566 UTM with a 5 mm/min crosshead motion rate and a 10 kN loading cell [42]. The specimens for tensile and flexural testing were printed using the recommended dimensions of the American Society for Testing and Materials (ASTM) standard. The tensile specimen was printed using the recommended dimensions of the tensile testing standard (ASTM D638). The specimen was set in the tensile jig fixture of the UTM. The crosshead motion of the UTM was 5 mm/min with a 100 N loading cell [37]. The ASTM D790 standard for flexural testing was implemented on the rectangular-shaped 3D-printed specimen. Each specimen was placed in a 3-point bending testing set of the UTM. The crosshead motion with a 5 kN loading cell was 10 mm/min [43].

For Fourier transform infrared spectroscopy (FTIR: Thermo Fisher Scientific, Waltham, MA, USA), a Thermo Fisher FTIR spectrometer equipped with an ATR (attenuated total reflectance) accessory was used to obtain the functional groups of the molecules in the SF sample. Sixty-four repetitive scans from 500 to 2500 cm^−1^ were averaged and presented.

For X-ray diffraction (XRD: Rigaku, Tokyo, Japan), the phase composit ion of the extracted HA powder and the HA within the composite PLA/PCL/HA filaments were characterized by XRD. The XRD chart was acquired with CuKα radiation (λ = 1.5418 Å) in the 2θ range of 20°–60°. 

For differential scanning calorimetry (DSC: Mettler Toledo DSC1 Star System, Columbus, OH, USA), to evaluate the effect of HA particles on the glass transition temperature and melting temperature of the PLA/PCL, the extruded composite filaments were examined at temperatures ranging from 25 to 200 °C. To check the consistency of the tests, the DSC experiments were run at least three times.

The morphology of the extruded filaments and printed specimens were then observed under a scanning electron microscope (SEM: JEOL JSM 6400, Tokyo, Japan).

For biodegradability testing, the specimens were printed in a square of 8 mm × 8 mm with a thickness of 2 mm. This design was printed for the SF coating, as well. The initial weight of each specimen was measured and recorded as *w_i_*. A phosphate-buffered saline (PBS) solution containing 1.6 µg/mL of lysozyme was prepared. The specimens were prepared for a 10 pieces/ratio/degradation period (90 pieces, total). The prepared specimens were placed in 24 well-plates with 1 mL of PBS containing lysozyme and kept in a 37 °C CO_2_ incubator for 7, 15, and 30 degradation days. At the end of days 7, 15, and 30 the specimens were rinsed with DI water and frozen in a −80°C freezer overnight. The frozen specimens were lyophilized for 48 h to obtain dried specimens. The weight of dried residual specimens was recorded as *w_f_*, and the percentage of weight loss was calculated using Equation (1) [32,44].

100 × ((*w_i_* − *w_f_*)/*w_i_*) (1)

For silk fibroin coating on the 3D-printed specimens, the printed specimens of all material ratios were placed in a 10 mm × 10 mm rectangular mold with a thickness of 3 mm. The SF sponge was mixed with the PBS solution (0.05 g/mL). Glutaraldehyde was used as a crosslinking agent for 0.0025% in the SF solution. Then, 200 µL of the preparing solution was dropped on each specimen in the rectangular mold. The specimens were completely covered by the solution. The mold containing the specimens with the SF solution was frozen in a −80 °C freezer overnight and lyophilized for 48 h. The specimens coated with SF were used for the biocompatibility test [45].

For biocompatibility testing, a human fetal osteoblast cell line (hFOB1.19, CRL NO.11372) was purchased from ATCC and expanded in DMEM/Ham’s F-12 medium (Sigma-Aldrich) supplemented with 10% fetal bovine serum (FBS), 100 U/mL penicillin, and 100 µg/mL streptomycin (basal media) until reaching cell confluence. Cells were seeded in 24-well plates at a density of 2 × 10^5^ cells/well and incubated at 37 °C with 5% CO_2_ for 24 h. The specimens consisted of PLA/PCL, PLA/PCL/5HA, and PLA/PCL/15HA and were divided into 2 groups: without an SF coating and with an SF coating. Each group was placed into the wells of the plates and incubated for 2 incubation periods (1 day and 3 days). At the indicated time of treatment, Alamar blue dye (10% *ν/ν* in PBS) was added to each well, and the plates were incubated at 37 °C for 4 h. Then, the condition media in each well was harvested, and the absorbances at 540 nm (test wavelength) and 620 nm (reference wavelength) were measured using a Titertek Multiskan M340 multiplate reader (ICN Flow, Costa Mesa, CA, USA) [44]. Each treatment was tested in triplicate. The formula for calculating the difference in the reduction percentage is shown in Equation (2):

% Cell viability = (OD of treated cell)/(OD of control cell) ∙100(2)

## 3. Results

### 3.1. Silk Fibroin Characterization

**Figure 3:**
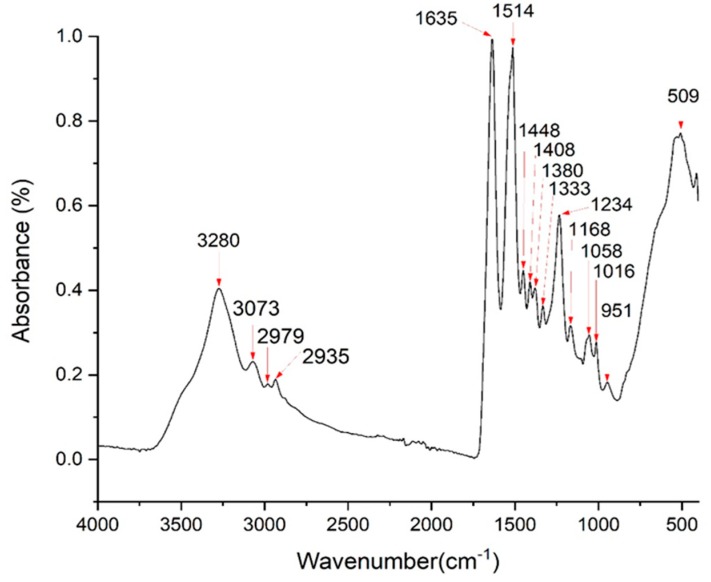
FTIR spectra of the silk fibroin (Luang Saraburi).

The extracted SF sponge from local Thai *Bombyx mori* (Luang Saraburi) silk cocoons was characterized using FTIR. The major conformation spectrum of the *Bombyx mori* SF structure consists of random coils (silk I) and β-sheets (silk II). The random coils showed strong absorption bands at 1625–1660 and 1640–1648 cm^−1^ (amide I), 1520–1545 cm^−1^ (amide II), and 1257–1258 cm^−1^ (amide III), and the β-sheets showed absorption bands at 1625–1640 cm^−1^ (amide I), 1520–1530 cm^−1^ (amide II), and 1219–1245 cm^−1^ (amide III) [45,46,47,48,49,50,51,52,53]. The intense amide band at 3291 cm^−1^ (beta-sheet) indicated NH bending [54]. The FTIR spectra of the extracted SF are shown in Figure 3. The indicated spectrum was similar to that of another study using the same extraction technique [54]. The FTIR peaks appeared within the recommended wavelengths. The peaks at 1635 cm^−1^, 1520 cm^−1^, and 1234 cm^−1^ represented amide I, amide II, and amide III, respectively. NH bending (beta-sheet) was indicated at 3280 cm^−1^.

### 3.2. Hydroxyapatite Characterization

**Figure 4:**
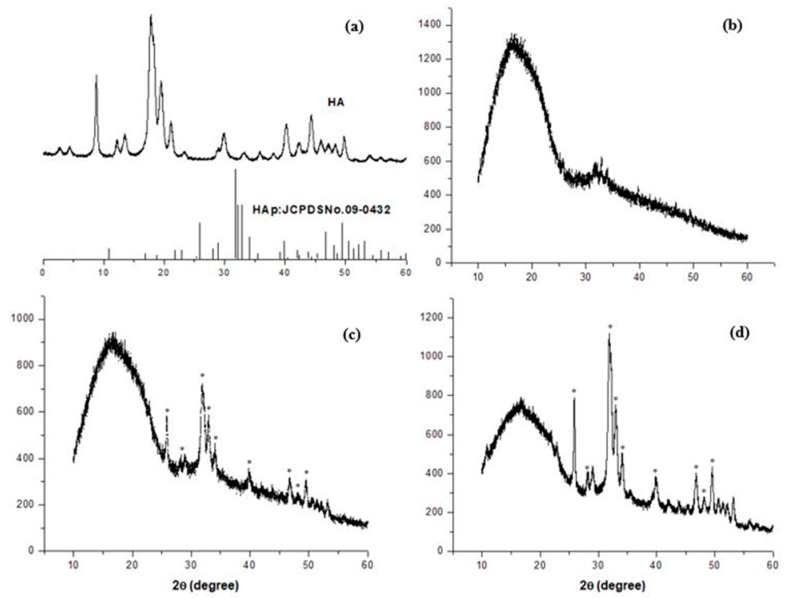
XRD patterns of (**a**) the locally extracted HA powder; (**b**) PLA/PCL, (**c**) PLA/PCL/5HA, and (**d**) PLA/PCL/15HA filaments.

XRD was performed on the locally extracted HA powder and the extruded filaments. The XRD pattern of the extracted HA is presented in Figure 4a. The pattern corresponds well to the JCPDS No. 9-432 data, which represents the pure HA pattern [55]. This comparison verified that the locally extracted HA consisted of a pure HA phase. The XRD patterns of the PLA/PCL, PLA/PCL/5HA, and PLA/PCL/15HA extruded composite filaments are shown in Figure 4b–d, respectively. The phase study showed that all HA peaks were contained in the composition filaments PLA/PCL/5HA and PLA/PCL/15HA; this phenomenon was more apparent in the sample with higher HA content because characteristic HA peaks were absent in the PLA/PCL filament. It can also be emphasized that during the filament extrusion process, no phase transformation occurred.

### 3.3. DSC Results

**Figure 5:**
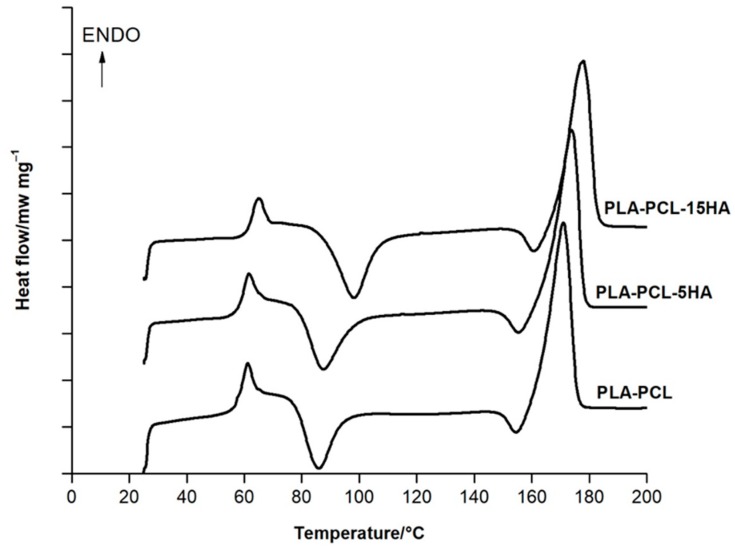
Differential scanning calorimetry (DSC) results of the extruded composite filaments.

To evaluate the effects of HA on the glass transition temperature (*T_g_*) and melting temperature (*T_m_*) of the PLA/PCL filaments, a dynamic DSC analysis was carried out on extruded filaments in a range of 25–200 °C. Figure 5 shows the DSC thermograms of the filaments of PLA/PCL, PLA/PCL/5HA, and PLA/PCL/15HA. The *T_g_* and *T_m_* of each composite’s filaments were measured using the peak temperature and inflection point as the measuring points. The findings are shown in Table 3; even with the highest HA content, the ceramic phase did not significantly affect the PLA/PCL *T_g_* and *T_m_*.

### 3.4. Filament Characterization

**Figure 6:**
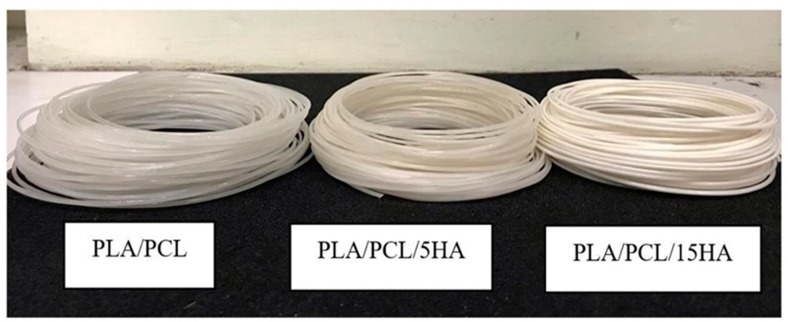
Appearance of the extruded composite filaments.

The diameters of the extruded composite materials were measured along the extrusion process to control for the required diameter (1.75 ± 0.05 mm). The appearance of extruded composite filaments is shown in Figure 6. The colors of the different filaments were visible. Filaments containing a lower HA powder percentage presented more transparency than did those with a higher HA powder percentage.

**Figure 7:**
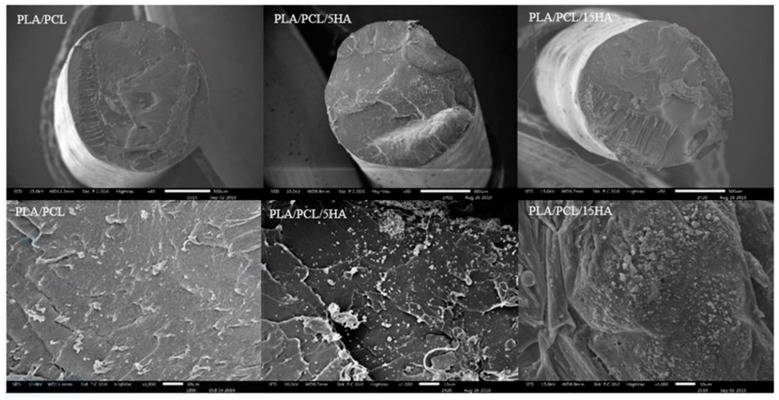
SEM images of the extruded filaments. Magnifications: 50× (Upper), 1000× (Lower).

Cross-sections of the extruded composite filaments were observed under SEM, as shown in Figure 7. The SEM images of PLA/PCL/5HA and PLA/PCL/5HA at 1000× magnification showed the presence of numerous small, bright particles uniformly distributed on the surface of the filaments.

### 3.5. Tensile Testing on the Extruded Filaments.

**Figure 8:**
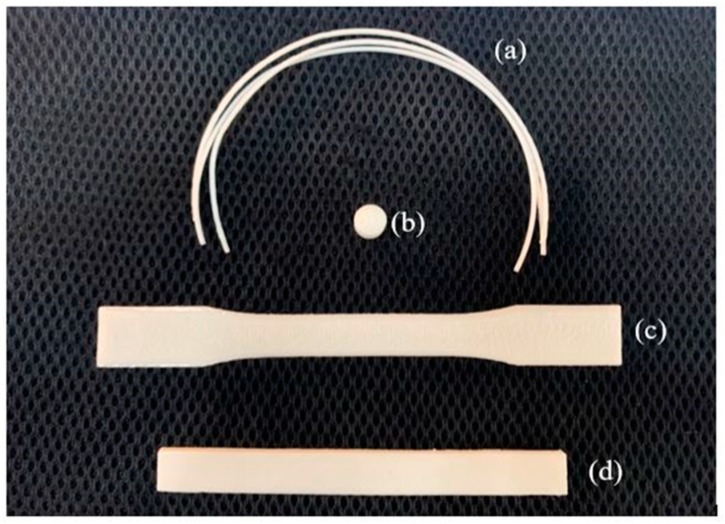
Filaments and specimens for mechanical testing, (**a**) filaments for tensile testing; (**b**) specimen for compression testing; (**c**) specimen for tensile testing; and (**d**) specimen for flexural testing.

**Figure 9:**
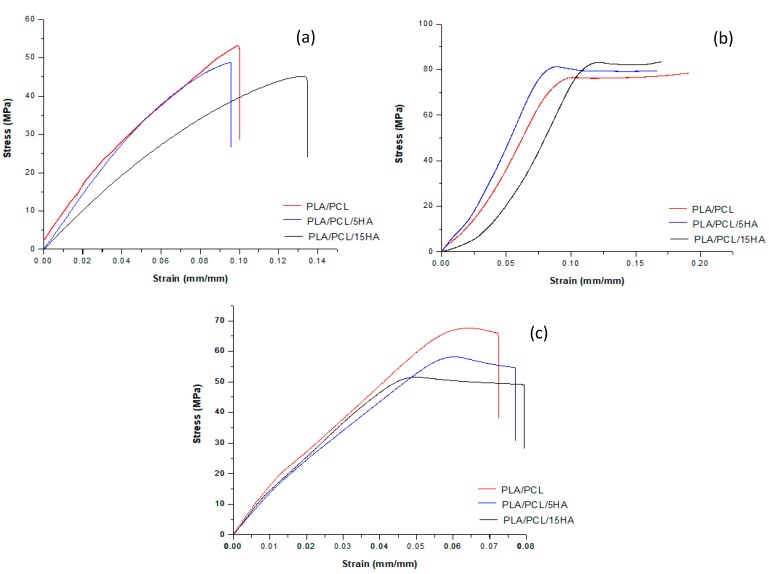
Representative stress–strain curve of the (**a**) filament tensile testing data; (**b**) specimen compression testing; and (**c**) specimen tensile testing for each composition.

Twenty-millimeter long extruded filaments were used for tensile testing (Figure 8a). Five filaments were randomly cut from a different region of the extruded filaments of each composition. Figure 9a shows the representative stress–strain curves from each of the filament compositions. The average results of the extruded filaments of each composition are presented in Table 4. The ultimate stress of the PLA/PCL/15HA filament was significantly lower than that of the other material compositions (45.73 ± 1.15 MPa). However, there was no difference in elongation at the break and modulus of elasticity for all groups.

### 3.6. Mechanical Testing the 3D-Printed Specimens

#### 3.6.1. Compression Testing of the 3D-Printed Specimens 

Compression tests were performed on the cylindrical specimens (Figure 8b). The stress–strain curves of the specimens for each composition were plotted and are presented in Figure 9b. A summary of the compression testing results consisting of ultimate strain, ultimate stress, and modulus of elasticity is shown in Table 5. Significantly higher compressive stress was present on the PLA/PCL/15HA specimen at 82.72 ± 1.76 MPa compared to the PLA/PCL specimen. However, the statistical analysis showed no significant differences between the PLA/PCL/5HA specimens compared to the other composition specimens. Therefore, only 15% HA powder added to the PLA/PCL matrix affected the ultimate stress of the compression test.

#### 3.6.2. Tensile Testing of the 3D-Printed Specimens 

The tensile specimen is shown in Figure 8c. Figure 9c shows the representative stress–strain curves for each composition. Table 6 presents a summary of all the mechanical properties that result from the tensile test. The summary table presents the average elongation at the break, the ultimate stress, and the modulus of elasticity of the specimens for each material composition. The highest elongation at break was 6.05% in the PLA/PCL/5HA specimen. The PLA/PCL specimen provided the highest ultimate stress and modulus of elasticity at 62.97 ± 4.31 MPa and 1.08 ± 0.07 GPa, respectively. The significantly lowest ultimate stress was observed in the PLA/PCL/15 specimen (52.05 ± 2.44 MPa) compared to the other composition specimens. The difference between the groups was similar to that of the tensile testing of the extruded filaments. The highest HA in the composite materials presented the lowest tensile stress.

#### 3.6.3. Flexural (Bending) Testing on the 3D-Printed Specimens

The rectangular specimens for all material compositions used in flexural (bending) testing are shown in Figure 8d. Table 7 compiles the average and standard division results for each specimen composition. No differences were observed among the PLA/PCL, PLA/PCL/5HA, and PLA/PCL/15HA printed specimens.

### 3.7. Biodegradability

**Figure 10:**
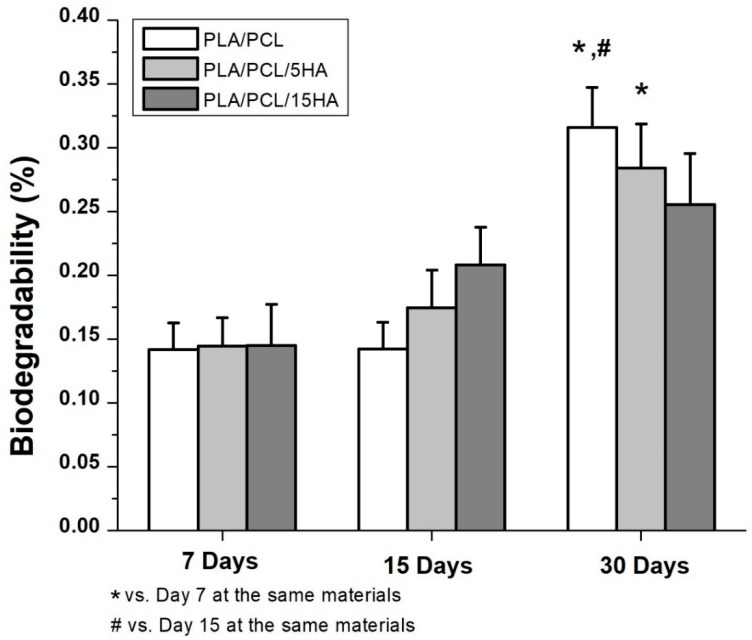
Biodegradation percentage of the composite specimens.

Biodegradability was obtained after soaking the specimens in a PBS solution containing lysozymes for 7, 15, and 30 days. The degradation percentages are presented as the mean ±SE (Figure 10). The comparison between day 7 and day 15 did not show any differences. Significantly different biodegradation was present in the specimens at day 30. A significantly higher degradation percentage was observed in the PLA/PCL specimen at day 30 at 0.32% ± 0.03% compared to days 7 and 15. In the PLA/PCL/5HA group, the degradation percentage of day 30 was significantly higher than that on day 7 only. There were no differences in the PLA/PCL/15HA group for any of the biodegradation periods.

In addition to the degradation observations, the obtained data can be used to predict degradation events, as shown in Equations (3)–(5). The percentage of degradation for the composition of each material was presented as *Y*_1_, *Y*_2_, and *Y*_3_, while the day of degradation was presented as *X*_1_, *X*_2_, and *X*_3_.

For the PLA/PCL 3D-printed sample, *Y*_1_ = 0.0434 *X*_1_ + 0.0696. (3)

For the PLA/PCL/5HA 3D-printed sample, *Y*_2_ = 0.0349 *X*_2_ + 0.0963. (4)

For the PLA/PCL/15HA 3D-printed sample, *Y*_3_ = 0.0115 *X*_3_ + 0.1898. (5)

### 3.8. Biocompatibility

**Figure 11:**
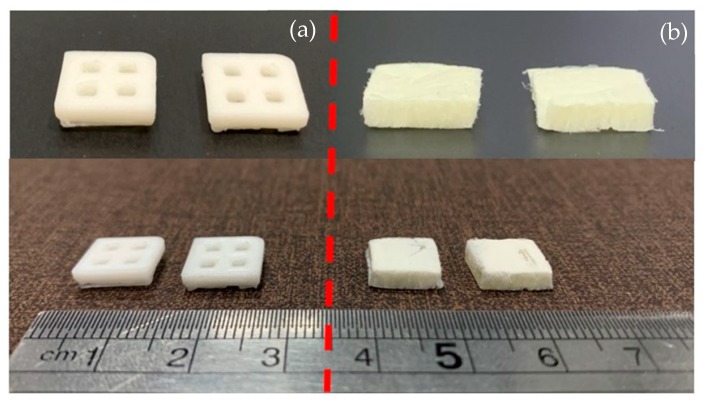
3D-printed specimen (**a**) without a silk fibroin (SF) coating and (**b**) with a SF coating.

**Figure 12:**
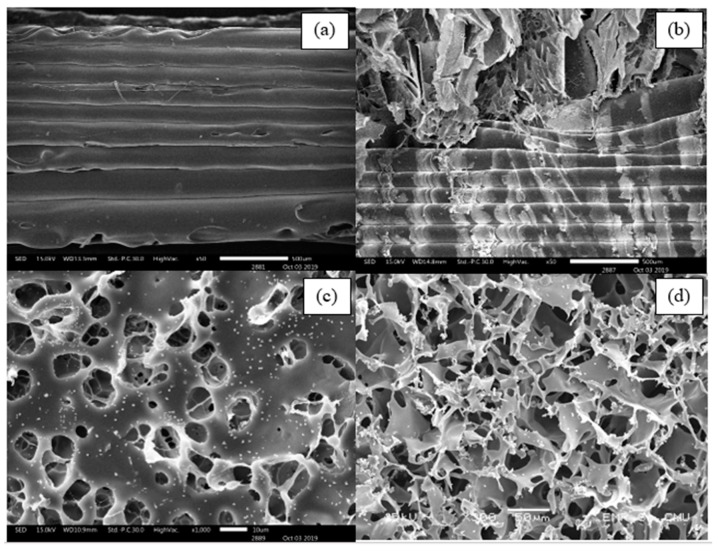
SEM images of the 3D-printed specimen for biological tests: (**a**) specimen without a SF coating, (**b**) specimen with a SF coating, (**c**) surface of the SF in the specimen cavity, and (**d**) bottom side of SF in the specimen cavity.

**Figure 13:**
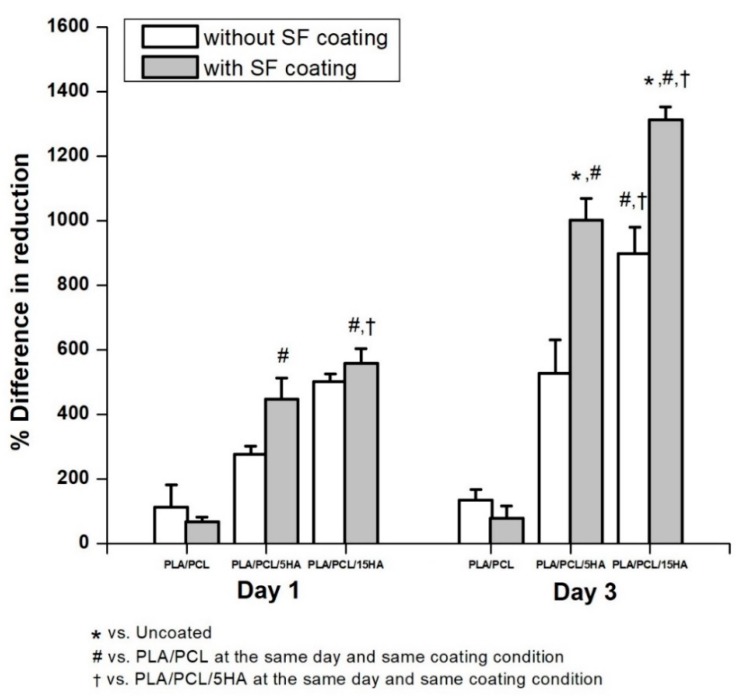
Effect of human fetal osteoblast cell proliferation, without a SF coating and with a SF coating.

The specimens for the biocompatibility test were divided into two groups: one with and one without SF coating. The specimens with and without SF coatings are presented in Figure 11. Under SEM, the visible surface of the 3D-printed specimen (Figure 12a) was covered by the SF structure (Figure 12b). The SF also filled the specimen cavities, as shown in Figure 12c,d. Each group consists of specimens with three different material compositions. An Alamar blue assay was performed on each group on days 1 and 3. A comparison of the collected absorbance between the control (cells only) and other groups is presented as the difference in the reduction percentage, as shown in Figure 13, which represents cell viability. On day 1, the results represented the non-cytotoxic ability of all the specimens. A comparison between the groups with and without the SF coating presented no difference. The SF coating group presented significantly higher cell viability for the PLA/PCL/15HA composition compared to the other compositions. The cell viability of PLA/PCL/5HA was also higher than that of the PLA/PCL in the SF coating group. However, the group of specimens without SF coating presented no difference on day 1. On day 3, the difference in the reduction percentage of the SF coating group was higher than that of the group without SF coating for PLA/PCL/5HA and PLA/PCL/15HA. The group of specimens without SF coating presented a significantly higher percentage for PLA/PCL/15HA. For PLA/PCL/15HA in the group with the SF coating, a significantly higher percentage was observed. Moreover, PLA/PCL/5HA presented a significantly higher percentage compared to PLA/PCL in the SF coating group.

## 4. Discussion

### 4.1. Filament Characterization

After the bioactive materials were characterized and compared to the standards, the composite biomaterial filaments were extruded. The first observed characteristic of the filaments was the diameter. Most commercial FFF 3D printers offer a 1.75 mm capability; thus, the extruder parameters were controlled to produce 1.75 ± 0.05 mm filaments [56,57]. Moreover, a 1.75 mm filament is flexible enough to spool and twist along the extrusion and printing process [50,51]. The presence of HA can be visibly recognized under SEM and confirmed using XRD, as shown in Figure 4. Determination of the critical composite material temperatures for both PLA and PCL was performed using DSC. The melting endotherms of PLA and PCL occurred from 165 to 180 °C [58] and from 63 to 66 °C [59,60], respectively. Figure 5 presents the two melting peaks of PLA/PCL in every composition ratio. Therefore, the combination of the composite materials did not affect the characteristics of the initial materials. However, the tension ability of the filaments was reduced due to the addition of HA. The addition of rigid particles, such as ceramics and metal powders, to the polymer decreased the tensile strength due to the agglomeration of particles in the polymer matrix without co-organized molecules between the particles and polymers [60,61]. Thus, a performance modification of this combination is still required to improve its tensile ability.

### 4.2. Specimen Characterization

The 3D-printed specimens were printed using extruded filaments of the recommended dimensions for each mechanical test. The results from the tensile test were related to the filament results [60,61]. The addition of HA decreased the tensile strength of the composite material specimens. In contrast, HA provided a higher compression ability to the 3D-printed specimens [62]. Basically, the plastic specimen’s tensile strength was approximately 66% of its compressive strength [63]. Therefore, the 3D-printed specimens can carry a higher compressive load than tensile load. The mechanical information on the related materials from previous studies was presented with our results, indicating that the composite polymers provide superior mechanical properties than the initial materials, as shown in Table 8.

### 4.3. Biological Properties

The biodegradation test results showed significant degradation at day 30 in the PLA/PCL group compared to that at day 7 and day 15, while at day 30 in the PLA/PCL/5HA group, significant differences were observed only compared to day 7. Furthermore, the PLA/PCL/15HA group showed no difference in degradation percentage. The greatest degradation was observed for PLA according to the highest degradation ability. The degradation caused by the hydrolysis of the polymer was mentioned in a previous study [74]. Water molecules cleaved the ester groups of the PLA, leading to oligomer and monomer release and resulting in a decrease in the molecular weight [14]. PLA can be either surface or bulk eroded [75] depending on the water diffusion rate and the hydrolysis reaction rate [14,76]. During degradation, tissue regeneration occurs and fills in the degraded cavities over time. Therefore, a biocompatible test is required. The targeted cells were used as an indicator of the possibilities of the fabricated composite materials. Several studies presented the ability of bioactive materials (HA and SF) to increase cell viability and cell proliferation [6,22,23,77]. The presence of HA activated the proliferation of bone cells, as predicted, because it is the mineral part of the bone extracellular matrix [78]. Moreover, the addition of SF in the specimens containing HA significantly increased the proliferation of bone cells.

## 5. Conclusions

The results show that the PLA/PCL/HA composite materials using FFF 3D printing have a high potential to fabricate interlocking nails for canine bone fracture treatment and indicate that the biological properties of the 3D-printed specimens can be increased when coated with local silk fibroin. Further tests, including a finite element analysis and the biomechanics of the FFF 3D-printed interlocking nails, are now ongoing to demonstrate other clinical requirements before an in vivo experiment.

## Figures and Tables

**Table 1:** Materials ratios and labeling.

Label	Materials ratio
PLA/PCL	70% PLA–30% PCL
PLA/PCL/5HA	(95% PLA/PCL)–5% HA
PLA/PCL/15HA	(85% PLA/PCL)–15% HA

**Table 2:** Controlled extrusion parameters of the desktop single-screw extruder.

Desktop Single-Screw Extruder Parameters	PLA/PCL	PLA/PCL/HA
Extrusion rotational speed (rpm)	65	30
Feed temperature (°C)	180	200
Nozzle temperature (°C)	180	190

**Table 3:** Glass transition temperature (*T_g_*) and melting point (*T_m_*) of the composite filaments, obtained from DSC analysis.

Sample	*T_g_* (°C)	*T_m_* (°C)
PLA/PCL	61.55	171.08
PLA/PCL/5HA	60.39	173.88
PLA/PCL/15HA	64.06	176.25

**Table 4:** Summary of the filament tensile testing.

Filament Composition	Mechanical Properties	Mean	SD
PLA/PCL	Elongation at Break (%)	10.21	2.53
	Ultimate Stress (MPa)	51.27	1.51
	Modulus of Elasticity (GPa)	1.23	0.18
PLA/PCL/5HA	Elongation at Break (%)	10.46	2.86
	Ultimate Stress (MPa)	49.02	2.13
	Modulus of Elasticity (GPa)	1.16	0.28
PLA/PCL/15HA	Elongation at Break (%)	12.40	5.08
	Ultimate Stress (MPa)	45.73 *^,#^	1.15
	Modulus of Elasticity (GPa)	1.20	0.33

* Statistically significant vs. PLA/PCL; # Statistically significant vs. PLA/PCL/5HA.

**Table 5:** Summary of compression testing.

Specimen Composition	Mechanical Properties	Mean	SD
PLA/PCL	Ultimate Strain (%)	9.22	1.92
	Ultimate Stress (MPa)	76.85	3.16
	Modulus of Elasticity (GPa)	1.02	0.04
PLA/PCL/5HA	Ultimate Strain (%)	9.13	2.37
	Ultimate Stress (MPa)	81.14	2.64
	Modulus of Elasticity (GPa)	1.23	0.34
PLA/PCL/15HA	Ultimate Strain (%)	10.74	1.24
	Ultimate Stress (MPa)	82.72 *	1.76
	Modulus of Elasticity (GPa)	1.06	0.14

* Statistically significant vs. PLA/PCL.

**Table 6:** Summary of the tensile testing results.

Specimen Composition	Mechanical Properties	Mean	SD
PLA/PCL	Elongation at Break (%)	5.77	0.88
	Ultimate Stress (MPa)	62.97	4.31
	Modulus of Elasticity (GPa)	1.08	0.07
PLA/PCL/5HA	Elongation at Break (%)	6.05	1.39
	Ultimate Stress (MPa)	57.47	2.01
	Modulus of Elasticity (GPa)	0.92	0.18
PLA/PCL/15HA	Elongation at Break (%)	5.61	0.39
	Ultimate Stress (MPa)	52.05 *^,#^	2.44
	Modulus of Elasticity (GPa)	0.98	0.10

* Statistically significant vs. PLA/PCL, # Statistically significant vs. PLA/PCL/5HA.

**Table 7:** Summary of bending testing.

Specimen Composition	Mechanical Properties	Mean	SD
PLA/PCL	Ultimate Strain (%)	11.21	1.88
	Ultimate Stress (MPa)	103.44	2.23
	Modulus of Elasticity (GPa)	2.40	0.41
PLA/PCL/5HA	Ultimate Strain (%)	11.35	3.57
	Ultimate Stress (MPa)	101.21	7.73
	Modulus of Elasticity (GPa)	2.39	0.77
PLA/PCL/15HA	Ultimate Strain (%)	11.28	1.47
	Ultimate Stress (MPa)	101.10	4.97
	Modulus of Elasticity (GPa)	2.34	0.38

**Table 8:** Mechanical properties of the materials.

Materials	Compressive Strength (MPa)	Tensile Strength (MPa)	Bending Strength (MPa)
**Previous Studies**			
Polylactic acid: PLA	18–93 [64]	46–66 [64]	97 [65]
Polycaprolactone: PCL	11.9–15.9 [66,67]	16 [68]	29 [65]
Hydroxyapatite: HA	174 [69]	50 [70]	-
Cortical bone	90–200 [71]	52 [70]	51 [70]
Cancellose bone	7–16 [72,73]	7.4 [70]	-
PLA filament	-	59.51 [40]	-
PLA 3D-Printed	85.72 ± 4.43 [42]	65.49 [40]	102.20 [40]
PLA/5HA 3D-Printed	86.03 ± 7.00 [42]	-	-
PLA/15HA 3D-Printed	85.66 ± 5.77 [42]	-	-
PLA/PCL/TiO_2_ 3D-Printed	-	45 [60]	-
**Current Study**			
PLA/PCL filament	-	51.27 ± 1.51	-
PLA/PCL/5HA filament	-	49.02 ± 2.13	-
PLA/PCL/15HA filament	-	45.73 ± 1.15	-
PLA/PCL 3D-Printed	76.85 ± 3.16	62.97 ± 4.31	103.44 ± 2.23
PLA/PCL/5HA 3D-Printed	81.14 ± 2.64	57.47 ± 2.01	101.21 ± 7.73
PLA/PCL/15HA 3D-Printed	82.72 ± 1.76	52.05 ± 2.44	101.10 ± 4.97
